# Increasing fish production in recirculating aquaculture system by integrating a biofloc-worm reactor for protein recovery

**DOI:** 10.1016/j.wroa.2024.100246

**Published:** 2024-08-02

**Authors:** Yuren Wang, Min Deng, Shuni Zhou, Lu Li, Kang Song

**Affiliations:** a Southern Marine Science and Engineering Guangdong Laboratory (Guangzhou), Guangzhou 511458, China; b State Key Laboratory of Freshwater Ecology and Biotechnology, Key Laboratory of Lake and Watershed Science for Water Security, Institute of Hydrobiology, Chinese Academy of Sciences, Wuhan 430072, China; c National-Regional Joint Engineering Research Center for Soil Pollution Control and Remediation in South China, Guangdong Key Laboratory of Integrated Agro-Environmental Pollution Control and Management, Institute of Eco-Environmental and Soil Sciences, Guangdong Academy of Sciences, Guangzhou 510650, China; d University of Chinese Academy of Sciences, Beijing 100049, China

**Keywords:** Tubificidae predation, Nitrogen mass balance, Community composition, Microbial function, Denitrification

## Abstract

Aquaculture, producing half of global fish production, offers a high-quality protein source for humans. Improving nitrogen use efficiency (NUE) through microbial protein recovery is crucial for increasing fish production and reducing environmental footprint. However, the poor palatability and high moisture content of microbial protein make its utilization challenging. Here, a biofloc-worm reactor was integrated into a recirculating aquaculture system (BW_RAS) for the first time to convert microbial protein into Tubificidae (Oligochaeta) biomass, which was used as direct feed for culturing fish. Batch experiments indicated that an aeration rate of 0.132 m^3^*L*^−1^*h*^−1^ and a worm density of 0.3 g cm^−2^ on the carrier were optimal for microbial biomass growth and worm predation, respectively. Compared to the biofloc reactor-based recirculating aquaculture system (B_RAS), the BW_RAS improved water quality, NUE, and fish production by 17.1 % during a 120-day aquaculture period. The abundance of heterotrophic aerobic denitrifier *Deinococcus* in BW_RAS was one order of magnitude higher than in B_RAS, while heterotrophic bacteria *Mycobacterium* was more abundant in B_RAS. Denitrifiers cooperated with organic matter degraders and nitrogen assimilation bacteria for protein recovery and gaseous nitrogen loss while competing with predatory bacteria. Function prediction and qPCR indicated greater aerobic respiration, nitrate assimilation, nitrification (AOB-*amoA*), and denitrification (*napA, nirK, nirS, nosZI*), but lower fermentation in BWR compared to BR. This study demonstrated that BW_RAS increased microbial protein production and aerobic nitrogen cycling through ongoing worm predation, further enhancing fish production to a commercially viable level.

## Highlights

• Aquaculture waste was transformed into Tubificidae biomass for direct feeding.
• Biofloc-worm reactor increased fish production by 17.1 % through protein recovery.
• Worm predation enhanced aerobic denitrifier *Deinococcus* by 11.7 times.
• Aerobic N cycling processes were increased, resulting in over 70 % gaseous N loss.

## Graphical abstract

**Figure:**
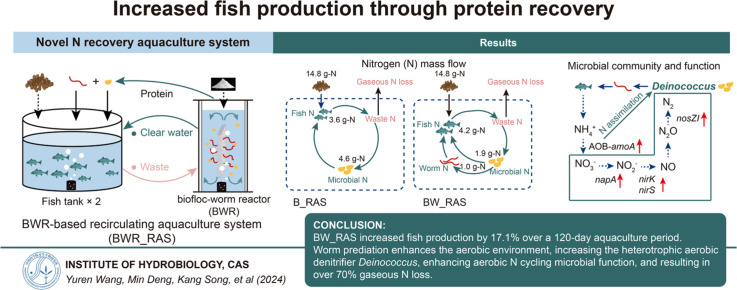

## 1 Introduction

With the global population expected to exceed 10 billion by 2050 and approximately 0.8 billion people currently afflicted by malnutrition, the escalating global food demand raises concerns about its ecological impact (El Abbadi and Criddle, 2019; Gentry et al., 2017). Aquaculture, the fastest-growing (8 % per year) animal protein industry, produces over 87.5 billion kilograms of fish annually, accounting for 49.2 % of global aquatic animal production (FAO, 2022). However, up to 75 % of nitrogen (N) input in aquaculture production is lost through water exchange and gas emissions, causing detrimental environmental impacts such as eutrophication, groundwater nitrate pollution, and greenhouse gas emissions (Hu et al., 2012; Lin and Lin, 2022). Specifically, China's aquaculture yielded ∼50 % (47 billion kilograms) of global aquaculture production, resulting in an annual N discharge of 1.6 billion kilograms (Wang et al., 2020). Hence, to meet global food demand and prevent excessive nitrogenous pollutants from entering the environment, feed N use efficiency (NUE) must increase to 70 % by 2050 (Wongkiew et al., 2018).

Microbial protein recovery, used in aquaculture waste treatment with microalgae, purple phototrophic bacteria, and biofloc technology, has proven to be cost-effective and environmentally friendly (Chun et al., 2018; Delamare-Deboutteville et al., 2019; Deng et al., 2021). Microbial protein can be used directly as a nutrient source for fish or harvested as a partial substitute for feed protein, thus increasing NUE (Delamare-Deboutteville et al., 2019). This aligns with the Sustainable Development Goals (https://sdgs.un.org/goals) and the blue transformation goal of aquaculture proposed by the United Nations (FAO, 2022). Due to the poor palatability of biofloc and high total suspended solid (TSS) in traditional in situ biofloc aquaculture system, only certain species like shrimp and tilapia can be cultured (Azim and Little, 2008). Recently, biofloc reactor-based recirculating aquaculture systems (B_RAS) were introduced to culture fish species that do not graze on biofloc directly (Azim and Little, 2008). These systems have improved N recovery efficiency to 47 % of feed N input through periodic microbial protein recovery (Deng et al., 2021). Nevertheless, the expensive dehydration process of biofloc and microalgae restricts the feasibility of recovering microbial protein as a feed protein substitute (Chun et al., 2018). Oligochaeta can utilize underutilized microbial protein, regulate biofloc biomass, and serve as a natural and appetizing feed for carnivorous, omnivorous, and ornamental fish species (Bouguenec, 1992; Helal et al., 2024). Many fish species in Western aquaculture do not accept live Oligochaeta meal. However, Oligochaeta biomass can be easily harvested and dehydrated as a feed protein substitute, offering an advantage over biofloc. The ideal operating conditions for biofloc-worm reactor (BWR) and their impacts on RAS performance and fish yield remain unclear.

**Fig. 1:**
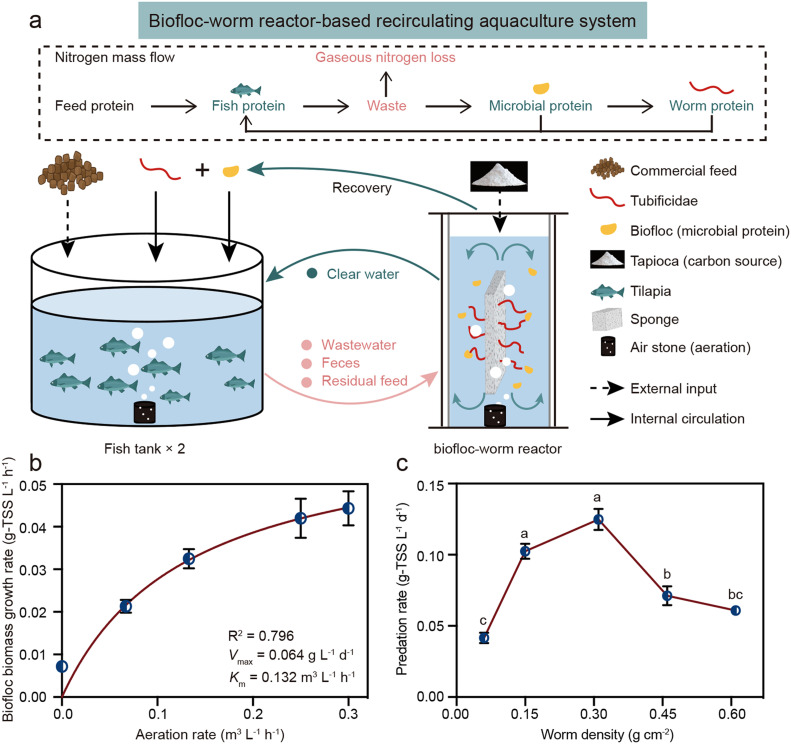
The novel biofloc-warm reactor-based recirculating aquaculture system (BW_RAS) and its optimal operational parameters. (a) Nitrogen mass flow and schematic diagram of BW_RAS. Effects of aeration rate and worm density for biofloc biomass growth rate (b) and worm predation rate (c), respectively. Apparent biofloc biomass growth rate affected by aeration rate was fit by the Michaelis-Menten equation.

While no studies have examined the use of Oligochaeta to recover biofloc biomass and increase fish production, several studies have explored their role in reducing waste sludge in wastewater treatment plants (Hendrickx et al., 2010; Zhang et al., 2020). Worm density and oxygen supply are critical design parameters for the worm reactor aimed at predating sludge (Hendrickx et al., 2010). Besides Oligochaeta, microbes play a crucial role in resource recovery and N cycling in this BWR (Fig. 1a). Denitrifiers are inevitably present in BWR, enhancing water quality but reducing the NUE of feed protein by converting nitrate to gaseous N (e.g., N_2_ and N_2_O), which is released into the atmosphere (Deng et al., 2021). Oligochaeta predation can significantly impact the microbial community composition, disproportionately reducing the abundance of slow-growing *K*-strategist bacteria compared to fast-growing *r*-strategist bacteria (Yu et al., 2020). The shift in microbial community structure should influence community function and, consequently, the NUE. However, the effects of Oligochaeta inoculation on microbial communities and their metabolic functions remain unclear.

This study aimed to enhance fish production by constructing a novel BWR-based RAS (BW_RAS). The optimal aeration rate for biofloc growth and the ideal worm density for predation were determined in batch experiments and then applied in the long-term BW_RAS. The performance of the BW_RAS was comprehensively compared to that of traditional B_RAS. 16S rRNA high-throughput sequencing and co-occurrence networks analysis were utilized to examine key species, their interactions, and their roles in the systems impacted by worm predation. Functional predictions and real-time quantitative polymerase chain reactions (qPCR) were performed to uncover changes in microbial function.

## 2 Results and discussion

### 2.1 Optimization of conditions for N recovery in the BWR

Previous studies identified aeration rate and worm density as key factors influencing biofloc biomass growth and worm predation, respectively (Hendrickx et al., 2009; Zhang et al., 2020). In the present study, the growth rates of biofloc biomass under various aeration rates were fitted using the Michaelis-Menten equation (Johnson, 1967) (Fig. 1b). Higher aeration rates can improve biofloc biomass growth rates (Fig. 1b) but require more energy (Sun et al., 2016). The increase in biofloc biomass growth rate slowed when the aeration rate exceeded the half-saturation constant (*K*_m_). Therefore, the *K*_m_ value (0.132 m^3^ *L*^−1^ *h*^−1^) was chosen as the optimal aeration rate for subsequent experiments.

The impact of worm densities on the predation rates of biofloc biomass is illustrated in Fig. 1c. Worm predation rates increased with higher densities but decreased once the density surpassed 0.3 g cm^−2^. The reduced biofloc biomass predation rate at higher worm densities was primarily due to limited growing space within the carrier (Tian et al., 2010). Finally, the optimal aeration rate and worm density (0.3 g cm^−2^) were implemented in the BW_RAS to evaluate performance during a long-term experiment.

### 2.2 Long-term and single-cycle performance of B_RAS and BW_RAS

**Fig. 2:**
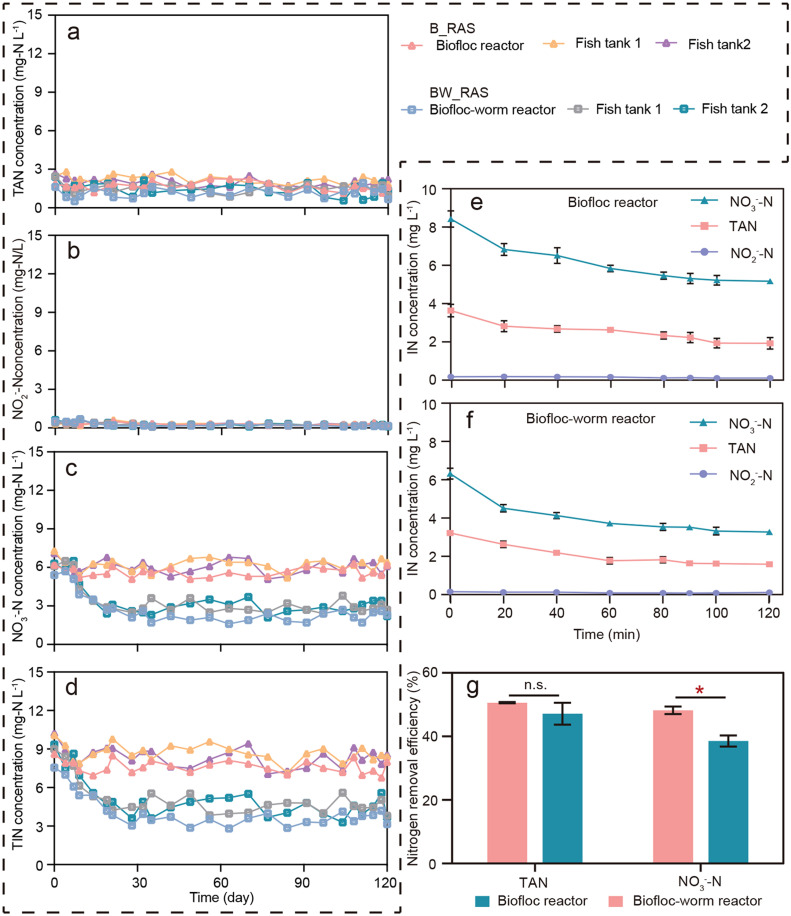
Long-term nitrogen species concentration in B_RAS and BW_RAS. TAN (a), NO_2_^−^-N (b), NO_3_^−^-N (c), and TIN (d) variation in reactors and fish tanks. Inorganic nitrogen concentration variations during a single cycle in biofloc reactor (e) and biofloc-worm reactor (f). (g) TAN and NO_3_^−^-N removal efficiency in biofloc reactor and biofloc-worm reactor.

The temperature, dissolved oxygen, and pH levels in all fish tanks were 25±0.4 ℃, 6.6 ± 0.3 mg *L*^−1^ and 7.8 ± 0.3, respectively, with no significant difference observed between the B_RAS and BW_RAS (*p* > 0.05) (Fig. S1). The variability of nitrogen species concentrations in B_RAS and BW_RAS over a long-term period of 120 days is shown in Fig. 2. Strict control of ammonia (NH_3_) and NO_2_^−^-N concentrations in the fish tank is necessary due to their toxicity to fish (Yogev and Gross, 2019). Total ammonia nitrogen (TAN) concentrations were significantly higher in fish tanks than in reactors for both B_RAS and BW_RAS (*p* < 0.05) (Fig. S2). This is because TAN primarily originates from fish excretion into water in fish tanks after feeding (Ip and Chew, 2010). Despite the high TAN concentration, the calculated NH_3_ concentration was 0.05±0.01 mg *L*^−1^, significantly below the average acute toxicity concentration for 32 freshwater fish species (2.8 mg NH_3_ *L*^−1^) (Randall and Tsui, 2002). Additionally, the NO_2_^−^-N concentrations in all fish tanks were maintained at safe levels (<0.28 mg *L*^−1^) (Fig. 2) (Kocour Kroupová et al., 2016). Hence, the water quality in both systems was suitable for tilapia culture.

Ammonia can undergo nitrification to form nitrite and eventually nitrate in both fish tanks and reactors (Han et al., 2020; Liu et al., 2019). In this study, the TAN and NO_2_^−^-N concentrations were maintained at 1.2 ± 0.3 mg *L*^−1^ and 0.2 ± 0.1 mg *L*^−1^, respectively, with no significant difference between B_RAS and BW_RAS (*p* > 0.05) (Fig. S2). However, the NO_3_^−^-N concentration in B_RAS was maintained at 5.7 ± 0.4 mg *L*^−1^, whereas in BW_RAS, it decreased significantly from 6.0 ± 0.5 mg *L*^−1^ to 2.9 ± 0.2 mg *L*^−1^ during the first 30-day and remained at 2.1 ± 0.4 mg *L*^−1^ from day 31 to day 120 (Fig. 2). Consequently, the total inorganic N (TIN) concentration in B_RAS was 1.9 times higher than that in BW_RAS after day 30. The NO_3_^−^-N can be denitrified to form N_2_ in the interior of biofloc or the worm gut, which is then lost to the atmosphere (Hu et al., 2014; Stief et al., 2009). The NO_3_^−^-N removal efficiency in BWR (48.2 ± 1.2 %) was significantly higher than in biofloc reactor (BR, 38.6 ± 1.7 %) (*p* < 0.05) (Fig. 2), resulting in significantly lower NO_3_^−^-N concentrations in BW_RAS compared to B_RAS (Fig. 2c).

### 2.3 Recovery of biofloc biomass and worm biomass from B_RAS to BW_RAS

**Fig. 3:**
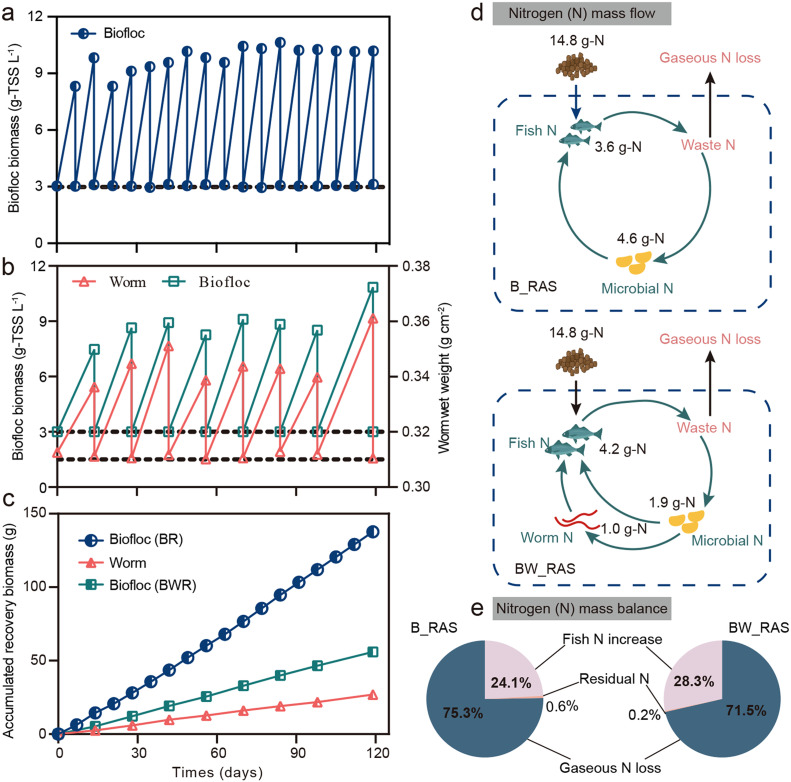
Biofloc biomass and worm biomass recovery performance. (a) Biofloc biomass was weekly recovery from biofloc reactor. (b) Biofloc biomass and worm biomass was biweekly recovery from biofloc-worm reactor. (c) Accumulated recovery amount of biofloc biomass and worm biomass from biofloc reactor (BR) and biofloc-worm reactor (BWR).

In this study, biofloc biomass and worm biomass were collected periodically (Fig. 3a, b). The average recovery rate of biofloc biomass in BWR (0.47±0.04 g day^−1^) was 40.5 % of that in BR (1.16 ± 0.12 g day^−1^), due to the conversion ratio of biofloc biomass into worm biomass (0.22±0.03 g day^−1^). In total, 137.6 g of biofloc biomass was recovered from BR, while 55.9 g of biofloc biomass and 26.9 g of worm biomass (wet weight) were recovered from BWR (Fig. 3c). The low recovered worm biomass in BWR was main because of low microbial N assimilation ratio (<25 %) of Tubificidae (Lou et al., 2022). These recovered biofloc and worm biomass were used to feed the fish in the tanks.

### 2.4 Growth performance of fish in B_RAS and BW_RAS

The growth performance of tilapia is summarized in Table S1. In BW_RAS, the average final fish weight was 10.3 % higher than in B_RAS (*p* < 0.001), resulting in a 25.9 g (17.1 %) greater increase in fish biomass (Table S1). The lower feed conversion ratio in BW_RAS indicated more efficient conversion of feed into fish weight (Mengistu et al., 2019). The feed conversion ratio in BW_RAS was exceptional compared to previous studies (Aboseif et al., 2022; Helal et al., 2024), highlighting the superior quality of Tubificidae biomass as feed for tilapia compared to biofloc biomass.

The N mass flow of B_RAS and BW_RAS is illustrated in Fig. 3d. In BW_RAS, the recovered N mass (1.9 g-N for biofloc biomass and 1.0 g-N for worm biomass) was only 63.0 % of that in B_RAS (4.6 g-N for biofloc biomass). However, the increase of fish N in BW_RAS exceeded that in B_RAS by 17.1 %. The N mass balance was further assessed (Fig. 3e). The increase in fish N in BW_RAS (28.3 %) outpaced that in B_RAS (24.1 %). Notably, the NUE in BW_RAS (28.3 %) was remarkable, as it typically ranges from 8.1 % to 27.1 % in biofloc-based tilapia aquaculture systems (Table S2). Furthermore, both RASs exhibited over 70 % gaseous N loss, indicating significant denitrification in both systems (Hu et al., 2014; Luo et al., 2023).

### 2.5 Microbial community composition

**Fig. 4:**
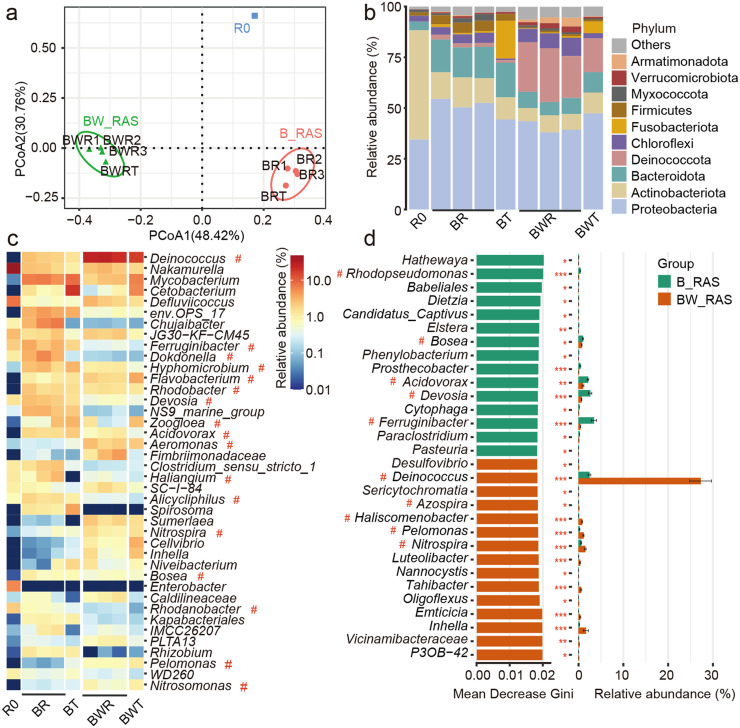
Microbial community composition in inoculated biofloc sample (R0) and both B_RAS and BW_RAS after 120-day culturing. (a) Principal coordinate analysis of the microbial community and (b) the microbial community structure at the phylum level. (c) Top 40 dominant genera in R0, B_RAS and BW_RAS. (d) Top 30 important genera for random forest classification in B_RAS and BW_RAS. # represent nitrifiers and denitrifiers. The top 30 taxa were assessed by the mean decrease Gini index, which represented the importance of each genus. Abbreviations: B_RAS, biofloc reactor based recirculating aquaculture system; BW_RAS, biofloc-worm reactor based recirculating aquaculture system; BR, biofloc reactor; BT, fish tank in B_RAS; BWR, biofloc-worm reactor; BWT, fish tank in BW_RAS.

High-throughput sequencing was employed to assess the impact of worm predation on the microbial community (Fig. 4). Both B_RAS and BW_RAS exhibited distinct microbial communities compared to the inoculated biofloc sample (R0) and each other (Fig. 4). Moreover, due to regular feeding of fresh biofloc from the reactor to the fish tanks, microbial community compositions in the fish tanks and reactor within the same RAS were similar (Fig. 4a). The Shannon and Simpson indices were significantly lower in BWR compared to BR (*p* < 0.01) (Table S3), indicating lower species diversity or evenness in BW_RAS (Wei et al., 2020). Overall, over 99.7 % of ASVs were classified at the phylum level (Fig. 4b). Actinobacteriota (53.8 %) and Proteobacteria (34.4 %) were the dominant phyla in R0. In B_RAS, the top three phyla were Proteobacteria (50.4 ± 4 %), Bacteroidota (15.9 ± 1 %), and Actinobacteriota (12.7 ± 1.6 %). In BW_RAS, they were Proteobacteria (42.1 ± 4 %), Deinococcota (22.1 ± 4.3 %), and Actinobacteriota (8.2 ± 1.6 %).

The microbial community was also analyzed at the genus level (Fig. 4c). Bacteria involved in N assimilation, nitrification, and denitrification are crucial for microbial protein recovery and gaseous N loss in RASs (Hu et al., 2014; Luo et al., 2023). Nitrifying bacterium *Nitrospira* was significantly more abundant in BWR than in BR (*p* < 0.05) (Fig. 4c, d). Moreover, worm predation on biofloc biomass significantly raised the relative abundance of *Deinococcus, Flavobacterium*, and *Aeromonas* in BWR compared to BR (Fig. S3). These genera include species with denitrifying capabilities (Zhang et al., 2018, 2024; Zhou et al., 2019). Specifically, *Deinococcus* was predominant in BW_RAS, with its relative abundance in BWR being 11.7 times higher than in BR. Species of genus *Deinococcus* can possess amylosucrase (Lee et al., 2024) and perform aerobic denitrification (Vinothkumar et al., 2021). Thus, heterotrophic *Deinococcus* can utilize tapioca and NO_3_^−^-N for growth and denitrification, consistent with the significantly higher NO_3_^−^-N removal efficiency observed in BWR (Fig. 2). On one hand, the increase in heterotrophic bacterial biomass (Figs. 4, S3) can supply microbial protein for Tubificidae, which is then consumed by fish. On the other hand, the enrichment of aerobic denitrifiers inevitably leads to significant gaseous N (e.g., N_2_ and N_2_O) loss (Fig. 3e). In contrast, heterotrophic bacterium *Mycobacterium* (He et al., 2021), aerobic organic matter degrader *Chujaibacter* (Zhang et al., 2022), and denitrifying bacterium *Dokdonella* (Wang et al., 2022) were significantly more abundant in BR than BWR (*p* < 0.05) (Figs. 4, S3). These bacteria stimulated biofloc biomass proliferation in BR, tripling its amount after one week of culture (Fig. 3a).

### 2.6 Co-occurrence networks analysis

**Fig. 5:**
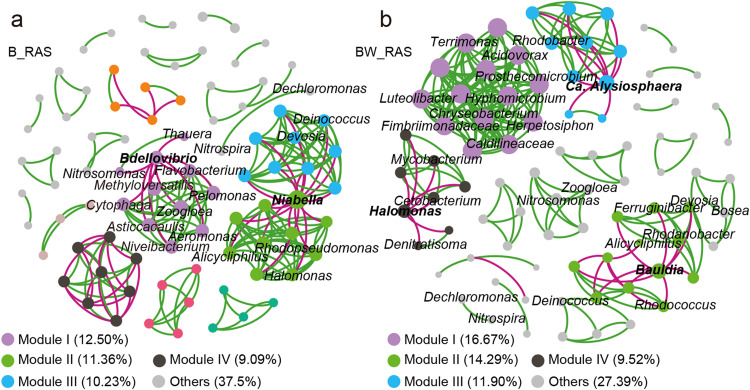
Co-occurrence networks of top 100 genera in microbial communities of (a) B_RAS and (b) BW_RAS, respectively. A connection stands for a strong (Spearman's ρ ≥ 0.6) and significant (Benjamini-Hochberg-adjusted p-value < 0.01) correlation. The genera were colored by modularity class. The size of each node is proportional to the number of connections (i.e., degree). The green connections indicate positive correlations, while green connections express negative correlations. Abbreviations: B_RAS, biofloc reactor based recirculating aquaculture system; BW_RAS, biofloc-worm reactor based recirculating aquaculture system.

Two co-occurrence networks were examined to investigate community assembly and reveal cooperative and competitive bacterial interactions in B_RAS and BW_RAS, respectively (Fig. 5). The actual networks showed higher modularity values and average clustering coefficients compared to the Erdös-Rényi random networks (Table S4), indicating strong interconnections among nodes. Green lines outnumbered red lines, suggesting prevalent positive correlations within these networks (Fig. 5, Table S4). In B_RAS, lignocellulose-degrading bacterium *Cytophaga* (Fan et al., 2022) and tapioca hydrolysis bacteria *Asticcacaulis* and *Niveibacterium* (Chun et al., 2016; Liu et al., 2005), were closely associated with nitrifying bacterium *Nitrosomonas* and denitrifying bacteria (*Thauera, Bdellovibrio, Flavobacterium, Methyloversatilis, Zoogloea, Aeromonas*) in module I.

Similarly, in BW_RAS, filamentous bacterium *Herpetosiphon* (Deng et al., 2021), organic matter degraders *Pedosphaeraceae* (Nguyen et al., 2023) and *Caldilineaceae* (Wu et al., 2023), and complex organic matter assimilating bacterium *Luteolibacter* (Yao et al., 2024) showed significant correlations with denitrifiers (*Terrimonas* (Zheng et al., 2019), *Acidovorax* (Tan et al., 2020), *Prosthecomicrobium* (Zhang et al., 2023), *Hyphomicrobium* (Zheng et al., 2019), Chryseobacterium (Tan et al., 2020), *Fimbriimonadaceae* (Chand et al., 2023)) in module I. Additionally, the synergy between *Pelomonas* and *Zoogloea* can remove NO_3_^−^-N via nitrate assimilation in module I (Tan et al., 2021). Moreover, nitrifier *Nitrospira* showed a positive correlation with the denitrifier *Dechloromonas* in both B_RAS and BW_RAS. Overall, heterotrophic hydrolytic bacteria and nitrifiers constructed a symbiotic network with denitrifiers to achieve N removal (Wu et al., 2023).

Notably, certain genera exhibited strong negative correlations with all other genera in dominant modules (Fig. 5). In B_RAS, naturally predatory denitrifier *Bdellovibrio* (Sockett, 2009; Wang et al., 2019) showed a notable negative correlation with all other bacteria in module I, while assimilatory nitrate reduction bacterium *Niabella* (Tian et al., 2024) exhibited significant negative correlations with all other bacteria in both module II and module III. In BW_RAS, filamentous bacterium *Candidatus Alysiosphaera* (Wongkiew et al., 2024) and heterotrophic bacterium *Bauldia* (Zheng et al., 2024) showed significant negative correlations with other denitrifiers in module II and module III, respectively. These findings suggest that heterotrophic denitrifiers engage in fierce competition with heterotrophic N assimilation bacteria and other predatory for N nutrients.

### 2.7 Microbial functional analysis

**Fig. 6:**
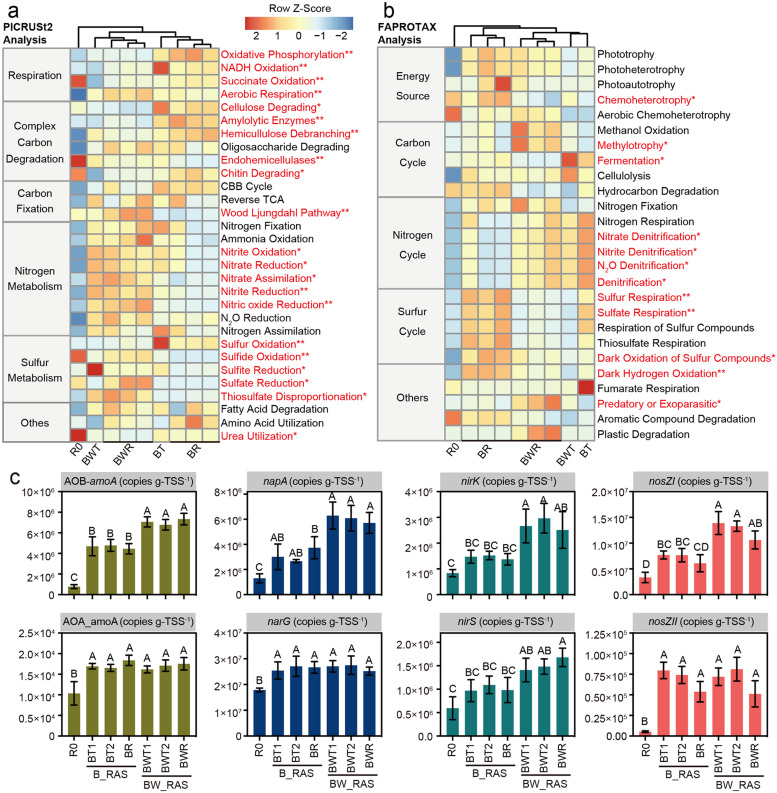
Metabolic pathways in different functional categories predicted from the 16S rRNA gene-based microbial compositions in inoculated biofloc sample (R0), fish tanks and reactor of B_RAS and BW_RAS. * and ^⁎⁎^ mean significant difference of predicted microbial function in BR and BWR at *p* < 0.05 and *p* < 0.01, respectively. RT-qPCR analysis of the nitrifying and denitrifying functional genes in inoculated biofloc and B_RAS and BW_RAS. Abbreviations: R0, inoculated biofloc samples; BT, fish tank in B_RAS; BR, biofloc reactor; BWT, fish tank in BW_RAS; BWR, biofloc-worm reactor. Different capital letters indicate significant difference at *p* < 0.05.

Both microbial functional prediction and qPCR were employed to investigate the impact of worm predation on the microbial community function (Fig. 6). The cluster analysis revealed significant differences in predicted functions between BR and BWR. Therefore, STAMP analysis was performed to compare the predicted microbial functions between BR and BWR (Fig. S4). Aerobic respiration (*p* < 0.01), nitrite oxidation (*p* < 0.05), denitrifying processes (nitrate, nitrite, and nitric oxide reduction), and nitrate assimilation (*p* < 0.05) in BWR were significantly higher compared to BR. FAPROTAX analysis indicated that BWR had stronger denitrification but reduced chemoheterotrophy and fermentation compared to BR (Fig. 6).

Quantitative analysis indicated a significant increase in all N cycling functional genes after the 120-day experiment (*p* < 0.05). The abundance of AOA-*amoA, narG*, and *nosZII* genes showed no significant difference between B_RAS and BW_RAS. However, the abundance of AOB-*amoA* and *napA* in BW_RAS was significantly higher than in B_RAS (*p* < 0.05). The fish tanks in BW_RAS exhibited a significantly higher abundance of *nirK* gene compared to the fish tanks in B_RAS (*p* < 0.05). Additionally, BWR had a significantly higher abundance of *nirS* gene compared to BR (*p* < 0.05). Furthermore, the abundance of *nosZI* in fish tanks and BWR of BW_RAS was significantly higher than in fish tanks and BR of B_RAS, respectively (*p* < 0.05). The AOB-*amoA* and *napA* genes are key functional genes involved in aerobic nitrification and denitrification, respectively (Xia et al., 2020). This aligns with the presence of a more aerobic environment (i.e., less biofloc biomass) in BW_RAS compared to B_RAS, attributed to worm predation (Fig. 3). The higher abundance of N cycling functional genes in BW_RAS than B_RAS was consistent with the higher abundance of nitrifiers and aerobic denitrifiers (e.g., *Deinococcus*) and higher NO_3_^−^-N removal efficiency in BW_RAS than B_RAS (Figs. 3, 4). Additionally, previous studies have shown high denitrification potential in the guts of aquatic macrofauna, especially filter- and deposit-feeders (Stief et al., 2009). The contribution of Tubificidae gut denitrification on N translation in BWR should be investigated in the future.

## 3 Conclusions

In this study, the biofloc biomass was predatory by Tubificidae, which was then recovered to feed culturing tilapia. That significantly increased fish production, which can reduce food-feed (e.g., forage fish and fish meal) competition, environmental pressure on arable land (producing plant-based replacement protein) and freshwater ecosystems, as well as reduce greenhouse gas emissions. The specific conclusions were provided below:

• BW_RAS increased NUE and 17.1 % of fish production during a 120-day aquaculture period.
• Worm predation on biofloc biomass increased the abundance of *Deinococcus* in BWR by 11.7 times compared to BR, potentially enhancing tapioca (carbon source) metabolic and denitrification.
• The N mass balance showed 71.5 %−75.3 % of gaseous N loss, consistent with the higher abundance of denitrifying functional genes in both RASs compared to the inoculated biofloc sample.
• Worm predation enhances the aerobic environment in BW_RAS compared to B_RAS, leading to increased aerobic ammonia oxidation (AOB-*amoA*) and denitrification (*napA, nirK, nirS, nosZI*). This ultimately results in a 9.6 % higher removal efficiency of NO_3_^−^-N in BWR compared to BR.

## 4 Materials and methods

### 4.1 Systems set-up and experimental operations

In the experiment setup, a 1.5-L BR (with a working volume of 1.2 L) was utilized to treat wastewater, residual feeds, and feces from two parallel 5.5-L fish tanks (each with a working volume of 4.8 L) in traditional B_RAS (Deng et al., 2021). For the BW_RAS, a 1.5-L BWR (with a working volume of 1.2 L) was employed to replace the BR in B_RAS (Fig. 1a). Prior to the start of the experiment, tilapia (*Oreochromis* sp.) were grown in the fish tanks for two weeks to acclimate to the environment. Each fish tank, culturing 20 tilapia with an mean weight of 1.5 ± 0.4 g, was initially stocked at a density of 6.3 kg *m*^−3^. The fish were fed commercial feed (Chenhui Feed Co. Ltd., Tianjin, China) with 35 % protein twice daily at 9:30am and 9:30pm. The daily feeding amount for each system was 1.2 ± 0.0 g, equivalent to 4 % of the initial fish biomass in each tank (Fig. S5). Occasionally, feeding was suspended due to the provision of biofloc and worm biomass, as well as the feeding behavior of the fish. For optimal inorganic nitrogen removal, mature biofloc biomass from a lab-scale aquaculture system was introduced into both the BR and the BWR to achieve an initial TSS concentration of 3.0 g *L*^−1^ (Deng et al., 2021). Additionally, tapioca starch was added as a supplementary carbon source to maintain a C/N (*w*/*w*) ratio of 20, ensuring a sufficient supply of organic carbon. The tapioca starch was added to the fish tanks daily at 9:30am.

Both the BR and the BWR were operated in 2.5-h cycles, each comprising a 1.5-h aeration phase followed by a 0.5-h settling phase. The treated clear water was then pumped into the fish tanks for 0.5 h using four peristaltic pumps (Kamoer, model NKCP-B08). Wastewater and solids (e.g., feces and residual feeds) overflowed from the fish tanks into these reactors. The hydraulic retention times (HRT) of the reactors and fish tanks were 5 h and 20 h, respectively. Both systems were located in an air-controlled room.

### 4.2 Effects of aeration rete and worm density on biofloc-worm reactor performance

Batch experiments were conducted to investigate the impact of aeration rate on biofloc biomass growth rate and the influence of worm (Oligochaeta, Tubificidae) density on its predation rate. Initially, 3 g *L*^−1^ of biofloc biomass was inoculated into a BR with a working volume of 1 L and cultured for 24 h at aeration rates of 0, 0.067, 0.130, 0.250, and 0.300 m^3^ l-water^−1^ *h*^−1^, respectively. The biomass growth rates were measured every 6 h for 24 h. Linear regression analysis was performed at different aeration rates to determine the biomass growth rates (Fig. S6). The oxygen concentrations required for worm growth were influenced by the aeration rates, and consequently, the aeration rates and biofloc biomass growth rates were fit using the Michaelis-Menten model (Wang et al., 2023). The half-saturation constant derived from the model was used as the optimal aeration rate for subsequent experiments.

The worms inoculated in a sponge (25 cm × 4 cm × 1 cm) were added to the BWR at five different densities (0.05, 0.15, 0.30, 0.45, and 0.60 g cm^−2^). Biofloc was then inoculated into the BWR with an initial concentration of 3 g-TSS *L*^−1^. No additional nutrients were added to the BWR, ensuring no external source of biofloc biomass. The BWR was maintained for 5 days, after which the final biofloc biomass was measured to determine the amount of biofloc biomass consumed. The optimal worm density was identified based on the treatment resulting in the highest biofloc biomass consumption.

### 4.3 Biofloc, worm, and fish biomass recovery

Biofloc biomass in the BR of B_RAS was measured and recovered weekly to maintain the optimal biofloc biomass level of 3 g *L*^−1^. Similarly, the biofloc and worm biomass in the BWR of BW_RAS were measured and recovered biweekly to ensure the optimal levels of biofloc (3 g *L*^−1^) and worm biomass (as mentioned above) were maintained. The recovered biofloc and worm biomass were used to feed the cultured fish in B_RAS and BW_RAS, respectively. At the end of the experiment, all cultured fish was weighted, and their growth performance was calculated (Deng et al., 2020). The N mass in the fish feed (*N*_feed_), biofloc biomass (*N*_biofloc_), worm biomass (*N*_worm_), fish biomass (*N*_tilapia_), and total nitrogen increase in water (*N*_water_) was calculated as previously reported (Deng et al., 2021; Yunjun and Yanling, 2004). Gaseous N loss was calculated using *N*_feed_ - *N*_tilapia_ - *N*_biofloc_ - *N*_worm_ - *N*_water_. Further details can be found in the Supporting Information.

### 4.4 Sample collection and water quality analysis

Water samples from fish tanks, BR, and BWR were collected biweekly to determine water quality. When the water quality was stable, additional water samples from the BR and the BWR were collected every 20 min during the 2-h working period of reactor to assess water quality variability. The dissolved oxygen concentration, pH, and temperature were measured in situ using an HQ30d YSI meter (YSI Inc., Yellow Spring, OH, U.S.A.). TAN, nitrate nitrogen (NO_3_^−^-N), and nitrite nitrogen (NO_2_^−^-N) concentrations were detected according to standard methods (APHA, 2012). The TIN concentration was calculated as the sum of TAN, NO_3_^−^-N, and NO_2_^−^-N concentrations. TAN includes ammonia (NH_3—_N) and ammonium (NH_4_^+^-N), which were calculated based on pH (see details in Supporting Information).

### 4.5 Microbial community, co-occurrence networks, and microbial function analysis

Nine representative microbial samples were collected from these systems, including the initial inoculated microbial sample (R0), final (triplicate) microbial samples from the BR (BR1, BR2, and BR3) and the BWR (BWR1, BWR2, BWR3), as well as two mixed samples (BT and BWT) from fish tanks in B_RAS and BW_RAS, respectively. DNA extraction was conducted using the DNeasy PowerSoil Kit (Qiagen, Hilden, Germany) (Deng et al., 2021). Illumina Miseq sequencing and analysis were performed to examine the microbial community structure (Deng et al., 2024). More details on Illumina Miseq sequencing, quality control assembly, amplicon sequence variants (ASVs) clustering, and taxonomy annotation are available in the Supporting Information. The rarefaction curve and high coverage index (> 0.998) indicated sufficient sequencing depth in this study (Fig. S7; Table S3). Co-occurrence network analysis was conducted for B_RAS and BW_RAS, respectively, to reveal the interactions among different genera (Deng et al., 2021). Additionally, PICRUSt2 (Douglas et al., 2020) and FAPROTAX (Deng et al., 2021) analyses were conducted to predict metabolic processes of microbial community. Furthermore, nitrifying (AOA-*amoA* and AOB-*amoA*), nitrate reduction (*napA* and *narG*), nitrite reduction (*nirS* and *nirK*), and N_2_O reduction (*nosZI* and *nosZII*) functional genes were assessed by qPCR (Table S5) (Deng et al., 2024).

### 4.6 Statistical analysis

The significance of long-term water quality variation between BR and BWR was determined using the paired-samples *t*-test. The effects of aeration rates on biofloc biomass growth rates, the effects of worm densities on biofloc biomass consumption, and the abundance of functional genes were examined using one-way analysis of variance (ANOVA) with Tukey's HSD test. The Michaelis-Menten model was fitted using Graphpad Prism (version 8.0.2). Principal coordinates analysis was performed using ‘ape’ and ‘ggplot2’ packages in R software (version 4.0.4). Additionally, significant differences in microbial functions and bacterial abundance between the BR and BWR were assessed using STAMP (version 2.1.3) software.

## CRediT authorship contribution statement

**Yuren Wang:** Writing – original draft, Visualization, Methodology, Formal analysis, Data curation. **Min Deng:** Writing – review & editing, Writing – original draft, Visualization, Funding acquisition, Formal analysis, Data curation, Conceptualization. **Shuni Zhou:** Writing – original draft, Formal analysis. **Lu Li:** Conceptualization. **Kang Song:** Supervision, Funding acquisition, Conceptualization.

## Declaration of competing interest

The authors declare that they have no known competing financial interests or personal relationships that could have appeared to influence the work reported in this paper.

## Data Availability

Data will be made available on request.

## Appendix Supplementary materials

Image, application 1
