# Supplementary material for: Increasing fish production in recirculating aquaculture system by integrating a biofloc-worm reactor for protein recovery
Source: Water Res X. 2024 Aug 2;24:100246. doi: 10.1016/j.wroa.2024.100246 (PMC11363497; doi:10.1016/j.wroa.2024.100246)
Supplement: Supplementary file 1 [file mmc1.pdf]

Supporting Information for

Increasing fish production in recirculating aquaculture system by integrating a biofloc-worm reactor for protein recovery

Yuren Wang[1,2,4,#], Min Deng[2,3,#], Shuni Zhou[2,4], Lu Li[1,2], Kang Song[2,4,*]

[1] Southern Marine Science and Engineering Guangdong Laboratory (Guangzhou), Guangzhou, 511458, China

[2] State Key Laboratory of Freshwater Ecology and Biotechnology, Institute of Hydrobiology, Chinese Academy of Sciences, Wuhan 430072, China

[3] National-Regional Joint Engineering Research Center for Soil Pollution Control and Remediation in South China, Guangdong Key Laboratory of Integrated Agro-environmental Pollution Control and Management, Institute of Eco-environmental and Soil Sciences, Guangdong Academy of Sciences, Guangzhou 510650, China

[4] University of Chinese Academy of Sciences, Beijing 100049, China

[#] Y.W. and M.D. contributed equally to this work.

* Corresponding authors: Kang Song (sk@ihb.ac.cn).

Phone: +86 027 68780523; Address: Donghu South road No. 7, Wuhan city, Hubei province, China

Text S1. Calculated methods

The carbon to nitrogen (C/N) ratio ($R_{C/N}$) of the commercial feed and tapioca added to the B_RAS and BW_RAS was calculated using the following equation:

$$R_{C/N} = (F \times PC \times 16\% \times 8.8 + S \times 82.8\% \times 44.4\%)/(F \times 35\% \times 16\%) \quad (1)$$

Where F is the feeding rate (g day^{-1}); PC is protein content in feed; 16% is the average nitrogen content of protein; 8.8 is the C/N ratio in the feeding commercial feed measured by Multi N/C 3100 TOC analyzer (Analytik Jena AG, Jena, Germany); S is the weight of daily tapioca addition; 82.8% is the carbohydrate in the commercial tapioca; and 44.4% is carbon content of tapioca.

Total ammonia nitrogen (TAN) contains un-ionized ammonia (NH_3-N) and ionized ammonia (NH_4^+-N) and only NH_3-N is toxic to fish. NH_3-N concentrations were calculated using the general equation of bases (Emerson et al., 1975):

$$NH_3 = (NH_3 + NH_4^+)/[1+10^{(pKa-pH)}] \quad (2)$$

Where pKa is based on the equation developed by Emerson et al. (1975): pKa = 0.09018 + 2729.92/T, (T in °K).

The N content of commercial feed into the B_RAS and BW_RAS was calculated according to the following equation:

$$N_{feed} = F_{total} \times 35\% \times 16\% \quad (3)$$

Where N_{feed} is total N mass in the fish feed (g); F_{total} is total feeding amount of fish feed, 35% is the average protein content of fish feed, 16% is the average nitrogen content of protein.

The N content of biofloc was calculated according to the following equation:

$$N_{biofloc} = DW_{biofloc} \times 21\% \times 16\% \quad (4)$$

Where $N_{biofloc}$ is total N mass in the biofloc; $DW_{biofloc}$ is the total dry weight harvest yield of biofloc (g), 35% is the average protein content of biofloc (Tubin et al., 2020), 16% is the average nitrogen content of protein.

The N content of tilapia was calculated according to the following equation:

$$N_{tilapia} = WW_{tilapia} \times 25\% \times 59\% \times 16\% \quad (5)$$

Where $N_{tilapia}$ is total N mass in the tilapia; $WW_{tilapia}$ is the wet weight of tilapia (g), 25% the dry matter content of tilapia, 59% is the average protein content of tilapia (Khanjani et al., 2021), 16% is the average nitrogen content of protein.

The N content of tubificidae was calculated according to the following equation:

$$N_{worm} = WW_{worm} \times 40\% \times 57\% \times 16\% \quad (6)$$

Where N_{worm} is total N mass in the tubificidae; WW_{worm} is the wet weight of tubificidae (g), 40% is the dry matter content of tubificidae, 57% is the average protein content of tubificidae (Yan et al., 2004), 16% is the average nitrogen content of protein.

Text S2. Library construction

DNA from all samples was amplified in triplicate by PCR for library construction. The 50-μL PCR mixture contained 25 μL of 2 × Premix Taq (Takara Biotechnology, Dalian Co. Ltd., China), 1 μL of each primer (10 μM), and 3 μL of template DNA (20 ng/μL). Additional ddH2O was added to reach a final volume of 50 μL. PCR was conducted using a Bio-Rad S1000 PCR thermal cycler (Bio-Rad Laboratory, CA, USA). The thermocycling steps were as follows: initial denaturation at 94°C for 5 minutes, followed by 30 cycles of 94°C for 30 seconds, 52°C for 30 seconds, and 72°C for 30 seconds, with a final extension at 72°C for 10

minutes. The quality of the library was assessed using a Qubit 2.0 Fluorometer (Thermo Fisher Scientific, MA, USA).

Text S3. Real-time Quantitative PCR

All specific primers for target genes were synthesized by Tyhygene Biotech Co., Ltd. (Wuhan, China). Each 20 μL reaction mixture consisted of 10 μL SYBR Premix (Takara, China), 0.5 μL DNA template, 0.5 μL each of forward and reverse primers, and 8.5 μL of sterile water. The forward and reverse primers for target genes, along with the annealing temperatures, are listed in Table S1. All plasmids containing the target genes were provided by Tyhygene Biotech Co., Ltd. (Wuhan, China) and subjected to a 10-fold serial dilution to serve as DNA templates for the construction of standard curves ($R^2 > 0.99$). RT-qPCR was performed using a CFX96 thermal cycler (Bio-Rad, USA). Relative abundance, defined as the absolute number of genes normalized to the weight of total suspended solid (TSS), was used in this study to compare the differences in denitrifying genes.

Text S4. Illumina Miseq Sequencing, Quality Control Assembly, Amplicon Sequence Variants (ASVs) Clustering, and Taxonomy Annotation

After extracting genomic DNA from the samples, the DNA of the conserved region was amplified using specific primers (341F/806R) with barcodes. The PCR products were gel-purified and quantified using a QuantiFluorTM fluorometer. Subsequently, the purified amplicons were equimolarly pooled for sequencing library construction. The library was paired-end sequenced (2×250) on an Illumina Miseq platform by Novogene (Beijing, China), following standard protocols.

The sequencing reads acquired in this study were analyzed using the QIIME 2 software package (version QIIME2-2023.5) (Bolyen et al., 2019). The paired-end reads underwent processing with Cutadapt (version 2.6) to eliminate primers and barcodes, yielding high-quality sequencing data (Martin et al., 2011). The "tools import" command was utilized to generate a qza file in the PairedEndFastqManifestPhred33V2 format. Following this, the "demux summarize" command was executed for visual inspection, and the distribution of sequence quality was assessed at https://view.qiime2.org. The QIIME 2 plugin "dada2 denoise-paired" was employed to denoise and cluster the sequences using the specified parameters: --p-trim-left-f 0, --p-trim-left-r 0, --p-trunc-len-f 240, --p-trunc-len-r 240 (Callahan et al., 2016). The SILVA database (silva-138-99-nb-classifier.qza) was trained for taxonomic classification, which was conducted using the "feature-classifier classify-sklearn" command (Mosley et al., 2022). Finally, the "tools export" command was employed to produce tables for ASVs and taxonomy.

Text S5. Code for Analysis of 16S rRNA Gene Sequences Using QIIME2

```
conda activate qiime2-2023.5

qiime tools import \
--type 'SampleData[PairedEndSequencesWithQuality]' \
--input-path manifest.txt \
--output-path paired-end-demux.qza \
```

```
--input-format PairedEndFastqManifestPhred33V2

qiime demux summarize \
--i-data paired-end-demux.qza \
--o-visualization paired-end-demux.qzv

qiime dada2 denoise-paired \
--i-demultiplexed-seqs paired-end-demux.qza \--o-table table.qza \
--o-representative-sequences rep-seqs.qza \
--o-denoising-stats stats.qza \
--p-trim-left-f 0 \
--p-trim-left-r 0 \
--p-trunc-len-f 240 \
--p-trunc-len-r 240 \
--p-n-threads 100
qiime vsearch cluster-features-de-novo \
--i-table table.qza \
--i-sequences rep-seqs.qza \
--p-perc-identity 0.97 \
--o-clustered-table table-dn-97.qza \
--o-clustered-sequences rep-seqs-dn-97.qza \
--p-threads 50

qiime tools export \
--input-path table-dn-97.qza \
--output-path output

biom convert -i output/feature-table.biom \
-o output/otu_table.tsv --to-tsv

qiime feature-classifier classify-sklearn \
--i-reads rep-seqs-dn-97.qza \
--i-classifier silva-138-99-nb-classifier.qza \
--o-classification taxonomy.qza

qiime tools export \
--input-path taxonomy.qza \
--output-path export_data
```

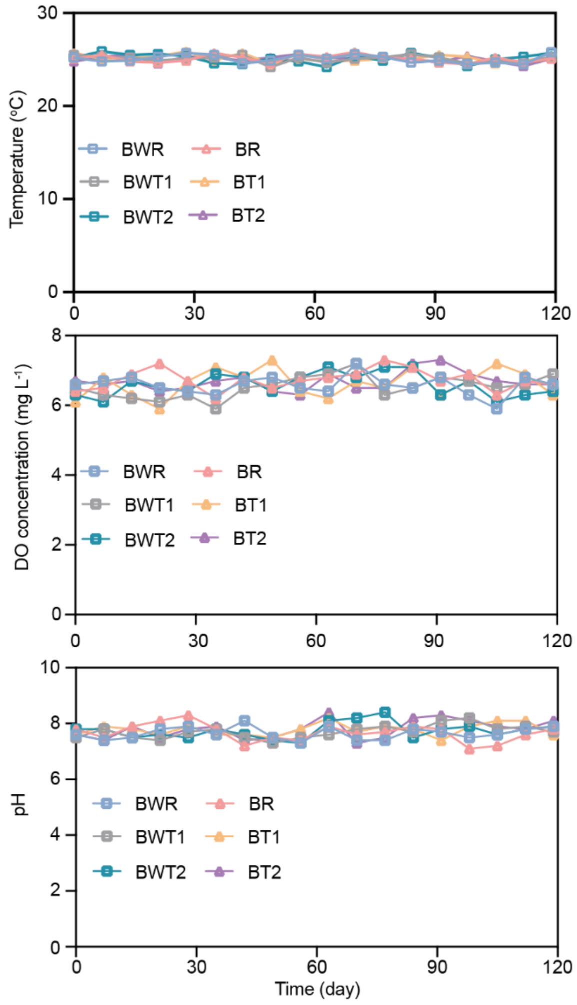

Figure S1. Long-term temperature, dissolved oxygen (DO) concentration, and pH in biofloc reactor-based recirculating aquaculture system (B_RAS) and biofloc-worm reactor-based recirculating aquaculture system (BW_RAS). BWR, biofloc-worm reactor; BR, biofloc reactor; BWT, fish tanks in BW_RAS; BT, fish tanks in B_RAS.

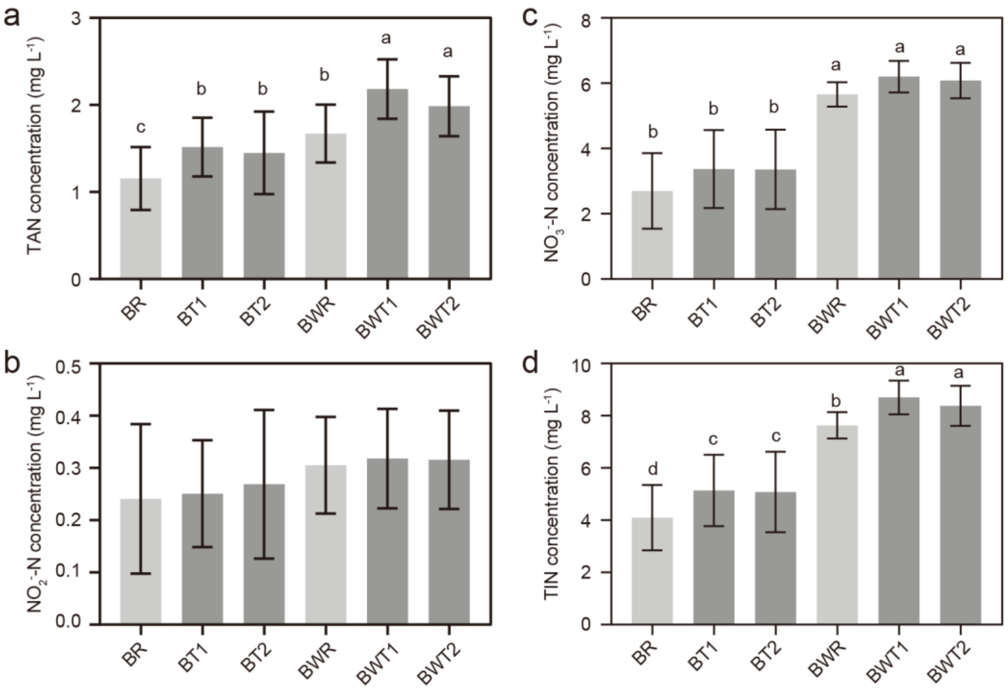

Figure S2. Average TAN (a), NO_2^--N (b), NO_3^--N (c), and (d) TIN concentrations in fish tanks and reactors in B_RAS and BW_RAS. BR, biofloc reactor; BT, fish tank in B_RAS; BWR, biofloc-worm reactor; BWT, fish tank in BW_RAS.

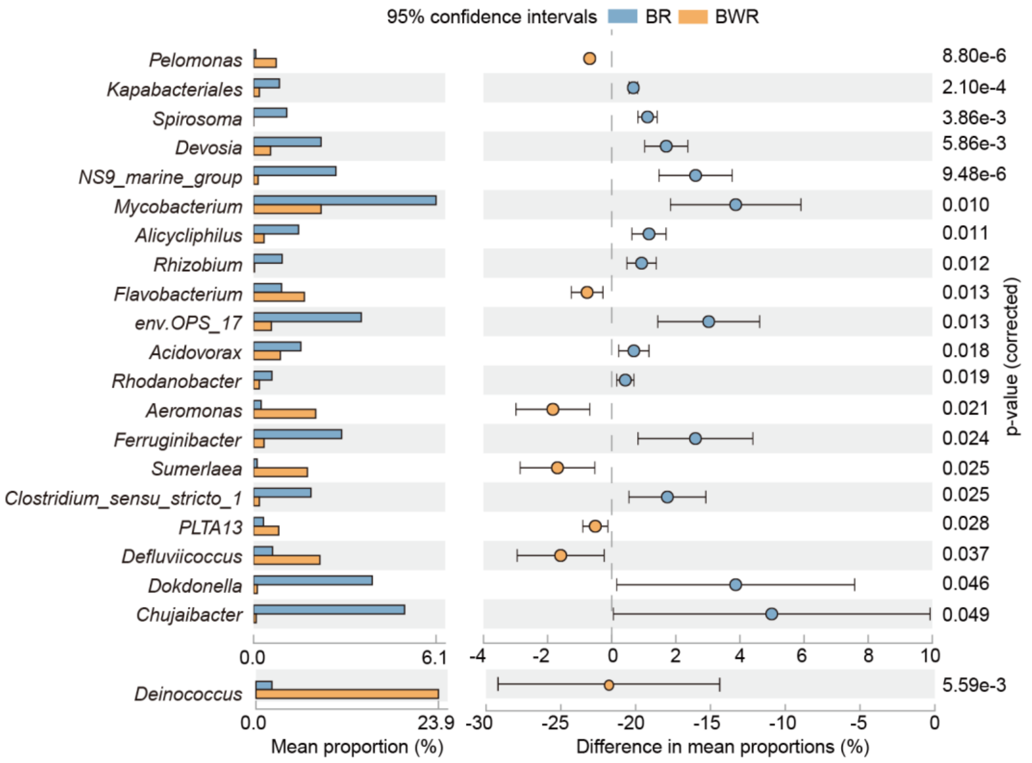

Figure S3. STAMP analysis of microbial community compositions in biofloc reactor (BR) and biofloc-worm reactor (BWR).

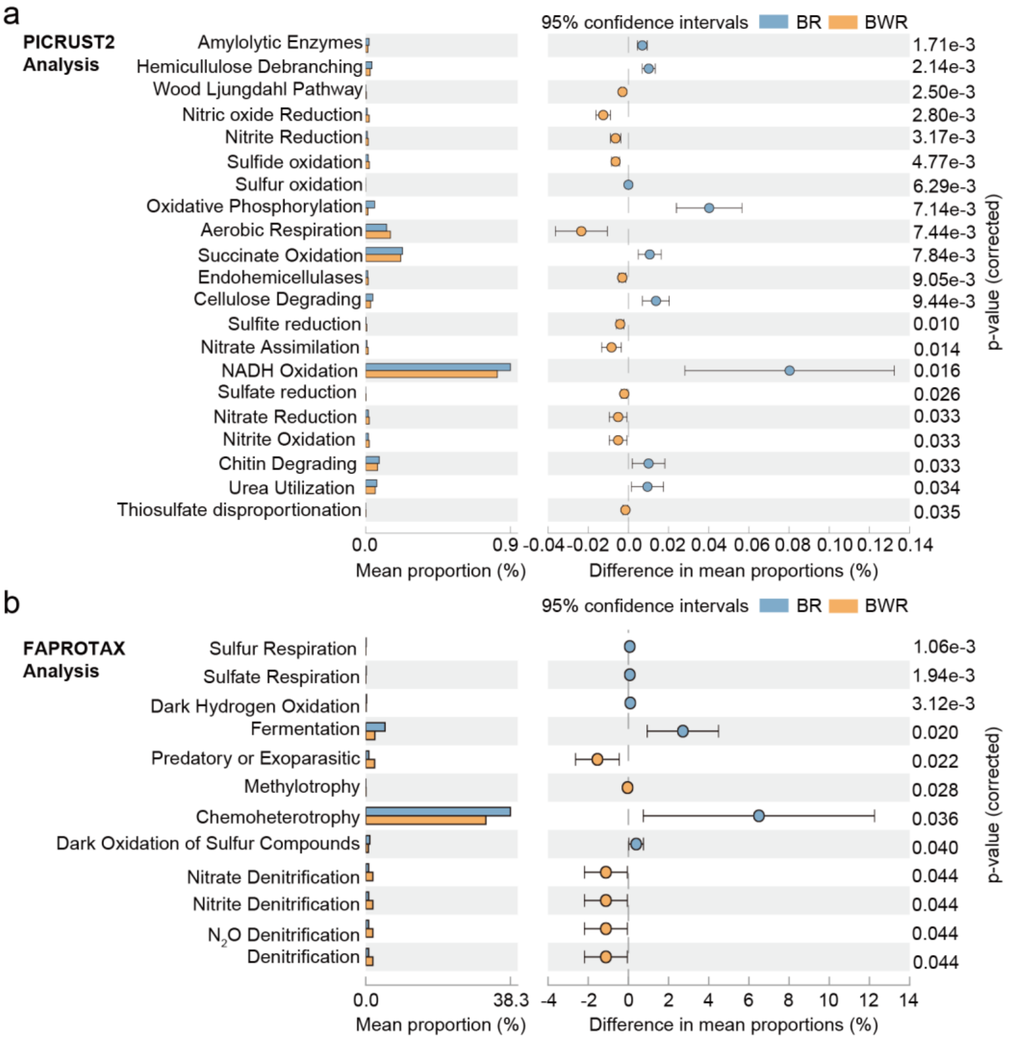

Figure S4. STAMP analysis reveals different microbial community function in biofloc reactor (BR) and biofloc-worm reactor (BWR) predicted by PICRUSt2 and FAPROTAX analysis.

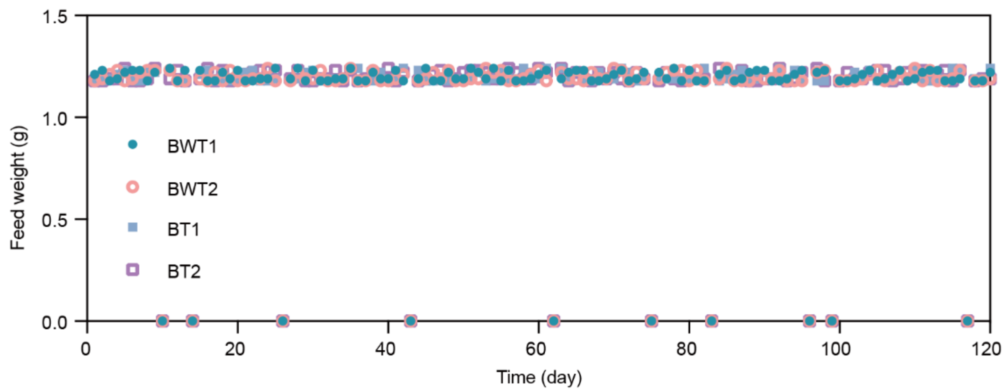

Figure S5. Daily feeding amount for each fish tanks.

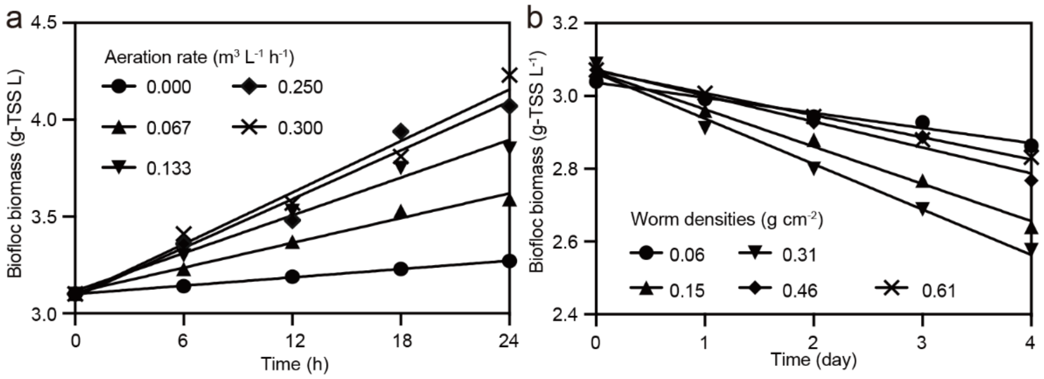

Figure S6. The impact of aeration rates and worm densities on biofloc biomass growth and worm predation, respectively. (a) Linear regression analysis of biofloc biomass growth under different aeration rates. (b) Linear regression analysis of biofloc biomass predation by worm under different worm densities. The slopes of linear regression represent the biofloc biomass growth rates and worm predation rates, respectively.

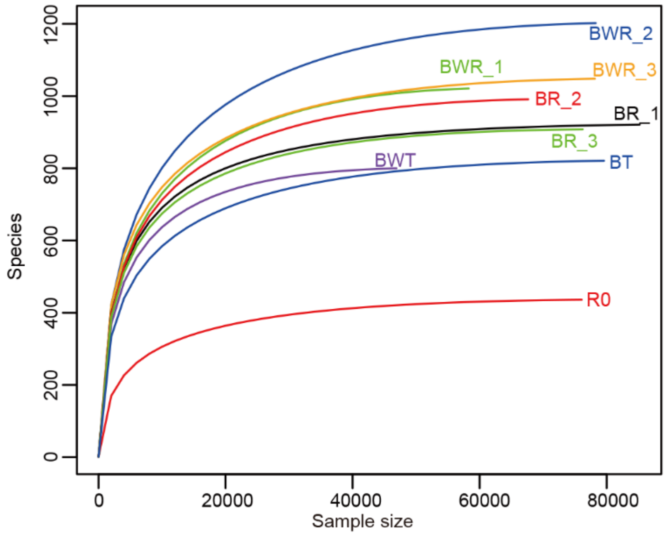

Figure S7. Rarefaction curve for microbial samples in inoculated sample (R0), fish tanks in B_RAS (BT), biofloc reactor in B_RAS (BR1, BR2, BR3), fish tanks in BW_RAS (BWT), and biofloc-worm reactor in BW_RAS (BWR1, BWR2, BWR3).

Table S1. Growth performance of tilapia reared in fish tanks of BRAS and BWRAS.

Parameters	B_RAS	BW_RAS
Average initial weight (n = 40) (g)	1.5±0.4	1.5±0.4
Average final weight (n =34) (g)	5.8±0.4	6.4±0.4***
Final stocking density (kg/m^3)	20.4±0.1	22.7±0.4
Survival rate (%)	85%	85%
Fish biomass increase (g)	151.2	177.1
Feed conversion ratio	1.8±0.1	1.5±0.0

Table S2. Water quality, feed nitrogen input, and fish growth performance in aquaculture system using biofloc technology.

System	TIN (mg/L)	TFN (g-N)	FNI (g-N)	FD (Kg/m^3)	NUE (%)	References
BAS	3.2	0.12	0.38	1.1	23.7	Ekasari et al. (2015)
BAS	7.38-47.9	13.02	1.06-1.87	1.5	8.1-14.3	García-Ríos et al. (2019)
BAS	9.24-9.75	142.4-427.3	30.5-80.8	1.0-3.0	15.8-21.4	Zaki et al. (2020)
BAS	31.4-39.8	45.5	9.6-10.9	2.9	21.1-24.0	Mabroke et al. (2021)
B_RAS	11.7	4.8	1.3	2.7	27.1	Mirzakhani et al. (2019)
BAS	1.93	13.1	3.3	0.5	25.2	de Sousa et al. (2019)
B_RAS	148.2	77.0	18.7	2.5	24.3	Martins et al. (2017)
B_RAS	14	75.8	17.0	26	22.4	Deng et al. (2020)
B_RAS	8.4-8.5	14.8	3.6	20.4	24.3	This Study
BW_RAS	3.68-3.81	14.8	4.2	22.7	28.4	This Study

Abbreviation: BAS, In situ biofloc aquaculture system; B_RAS, biofloc reactor-recirculating aquaculture system; BW_RAS, biofloc-worm reactor-based recirculating aquaculture system; TIN, total inorganic nitrogen; TFN, total feed nitrogen input; FNI, fish nitrogen increase; FD, Final density; NUE, nitrogen use efficiency.

Table S3. Alpha diversity of microbial community in inoculated biofloc, biofloc reactor, biofloc-worm reactor, and fish tanks in B_RAS and BW_RAS, respectively.

	Observed ASVs	Chao1	ACE	Shannon	Simpson	Coverage
R0	409.0	427.3	432.1	3.39	0.90	1.000
BR	879.7 ± 49.7	913.0 ± 53.5	914.7 ± 53.4	5.23 ± 0.07**	0.99 ± 0.00**	0.998 ± 0.000
BWR	1003.7 ± 70.4	1061.2 ± 84.6	1064.5 ± 87.9	4.80 ± 0.12	0.94 ± 0.01	0.998 ± 0.001
BT	752.0	792.6	792.5	4.78	0.97	0.998
BWT	778.0	795.4	797.2	4.92	0.97	1.000

R0, inoculated biofloc sample; BWR, biofloc-worm reactor; BR, biofloc reactor; BWT, mixed fish tank samples in BW_RAS; BT, mixed fish tank samples in B_RAS. **: $p < 0.01$.

Table S4. Topological Properties of Real Co-occurrence Networks and Random Networks

Network metrics	B_RAS	BW_RAS
	Real Networks	
no. of nodes	87	84
no. of edges	205	215
average path length	1.329	1.267
graph density	0.042	0.062
network diameter	2	4
average clustering coefficient	0.978	0.967
average degree	4.12	5.119
modularity	0.801	0.767
	Random Networks	
average path length	3.002 ± 0.036	2.855 ± 0.030
graph density	0.0548 ± 0	0.062 ± 0
network diameter	6.075± 0.538	5.648± 0.573
average clustering coefficient	0.054± 0.013	0.061 ± 0.012
average degree	4.713 ± 0	5.120 ± 0
modularity	0.371 ± 0.023	0.350 ± 0.023

Table S5. Primers used for real-time quantitative PCR analysis of *narG*, *napA*, *nirK*, *nosZ*.

Target gene	Primer	Primer sequence (5' - 3')	Annealing temperature (℃)	Amplification size (bp)	Reference
AOA-amoA	Arch-amoAF	STAATGGTCTGGCTTAGACG	53	635	Francis et al.,2005
	Arch-amoAR	GCGGCCATCCATCTGTAT GT			
AOB-amoA	amoA-1F	GGGGTTTCTACTGGTGGT	54	491	Rotthauwe et al.,1997
	amoA-2R	CCCCTCKGSAAAGCCTTCTTC			
narG	narG-f	TCGCCSATYCCGGCSATGTC	58	173	Bru et al., 2007
	narG-r	GAGTTGTACCAGTCRGCSGAYTCSG			
napA	V17m	TGGACCATGGGCTTCAACC	61	152	Bru et al., 2007
	napA4r	ACCTCGCGCGCGGTGCCGCA			
nirK	nirKFlaCu	ATCATGGTSCTGCCGCG	55	473	Chen et al., 2017
	nirKR3Cu	GCCTCGATCAGRTTGTGGTT			
nirS	nirScd3aF	GTSAACGTSAAGGARACSGG	55	426	Chen et al., 2013
	nirS-R3cd	GASTTCGGRTGSCTCTTGA			
nosZ I	nosZ2F	CGCRACGGCAASAAGGTSMSSGT	60	276	Henry et al. 2006
	nosZ2R	CAKRTGCAKSGCRTGGCAGAA			
nosZ II	nosZ-II-F	CTIGGICCIYTKCAYAC	54	690	Jones et al.,2013
	nosZ-II-R	GCIGARCARAAITCBGTRC			

Supporting References

1. Bolyen, E., Rideout, J.R., Dillon, M.R., Bokulich, N.A., Abnet, C.C., Al-Ghalith, G.A., et al., 2019. Reproducible, interactive, scalable and extensible microbiome data science using QIIME 2. Nat. Biotechnol. 37, 852-857.
2. Bru, D., Sarr, A. and Philippot, L., 2007. Relative abundances of proteobacterial membrane-bound and periplasmic nitrate reductases in selected environments. Appl. Environ. Microbiol. 73(18), 5971-5974.
3. Callahan, B.J., McMurdie, P.J., Rosen, M.J., Han, A.W., Johnson, A.J.A. and Holmes, S.P., 2016. DADA2: High resolution sample inference from Illumina amplicon data. Nat. Methods 13, 581-583.
4. Chen, R., Deng, M., He, X. and Hou, J., 2017. Enhancing nitrate removal from freshwater pond by regulating carbon/nitrogen ratio. Front. Microbiol. 8, 1712.
5. de Sousa, A. A., Pinho, S. M., Rombenso, A. N., de Mello, G. L. and Emerenciano, M. G. C., 2019. Pizzeria by-product: a complementary feed source for Nile tilapia (*Oreochromis niloticus*) raised in biofloc technology? Aquaculture 501, 359-367.
6. Deng, M., Li, L., Dai, Z., Senbati, Y., Song, K. and He, X., 2020. Aerobic denitrification affects gaseous nitrogen loss in biofloc-based recirculating aquaculture system. Aquaculture 529, 735686.
7. Ekasari, J., Rivandi, D. R., Firdausi, A. P., Surawidjaja, E. H., Zairin Jr, M., Bossier, P. and De Schryver, P., 2015. Biofloc technology positively affects Nile tilapia (*Oreochromis niloticus*) larvae performance. Aquaculture 441, 72-77.
8. Francis, C.A., Roberts, K.J., Beman, J.M., Santoro, A.E. and Oakley, B.B., 2005. Ubiquity and diversity of ammonia oxidizing archaea in water columns and sediments of the ocean. Proc. Natl. Acad. Sci. U.S.A. 102, 14683–14688.
9. García-Ríos, L., Miranda-Baeza, A., Coelho-Emerenciano, M. G., Huerta-Rábago, J. and A.Osuna-Amarillas, P., 2019. Biofloc technology (BFT) applied to tilapia fingerlings production using different carbon sources: Emphasis on commercial applications. Aquaculture 502, 26-31.
10. Henry, S., Bru, D., Stres, B., Hallet, S. and Philippot, L., 2006. Quantitative detection of the *nosZ* gene, encoding nitrous oxide reductase, and comparison of the abundances of 16S rRNA, *narG*, *nirK*, and *nosZ* genes in soils. Appl. Environ. Microbiol. 72, 5181-5189.
11. Hu, Z., Lee, J.W., Chandran, K., Kim, S. and Khanal, S.K., 2012. Nitrous oxide (N_2O) emission from aquaculture: a review. Environ. Sci. Technol. 46, 6470-6480.
12. Jones, C.M., Graf, D.R.H., Bru, D., Philippot, L. and Hallin, S., 2013. The unaccounted yet abundant nitrous oxide-reducing microbial community: a potential nitrous oxide sink. ISME J. 7, 417–426.
13. Khanjani, M. H., Alizadeh, M., Mohammadi, M. and Sarsangi, A. H., 2021. Biofloc system applied to Nile tilapia (*Oreochromis niloticus*) farming using different carbon sources: growth performance, carcass analysis, digestive and hepatic enzyme activity. Iran. J. Fish. Sci. 20 (2), 490–513
14. Mabroke, R. S., Zidan, A. E. N. F., Tahoun, A. A., Mola, H. R., Abo-State, H. and Suloma, A., 2021. Feeding frequency affect feed utilization of tilapia under biofloc system condition during nursery phase. Aquacult Rep 19, 100625.
15. Martin, M., 2011 Cutadapt removes adapter sequences from high-throughput sequencing

reads. EMBnet.journal. 17, 10-12.
16. Martins, G. B., Tarouco, F., Rosa, C. E. and Robaldo, R. B., 2017. The utilization of sodium bicarbonate, calcium carbonate or hydroxide in biofloc system: water quality, growth performance and oxidative stress of Nile tilapia (*Oreochromis niloticus*). Aquaculture 468, 10-17.
17. Mirzakhani, N., Ebrahimi, E., Jalali, S. A. H. and Ekasari, J., 2019. Growth performance, intestinal morphology and nonspecific immunity response of Nile tilapia (*Oreochromis niloticus*) fry cultured in biofloc systems with different carbon sources and input C: N ratios. Aquaculture 512, 734235.
18. Mosley, O.E., Gios, E., Close, M., Weaver, L., Daughney, C. and Handley, K.M., 2022. Nitrogen cycling and microbial cooperation in the terrestrial subsurface. ISME J. 16, 2561-2573.
19. Rotthauwe, J.H., Witzel, K.P. and Liesack, W., 1997. The ammonia monooxygenase structural gene *amoA* as a functional marker: molecular fine-scale analysis of natural ammonia-oxidizing populations. Appl. Environ. Microb. 63, 4704–4712.
20. Tubin, J. S. B., Paiano, D., de Oliveira Hashimoto, G. S., Furtado, W. E., Martins, M. L., Durigon, E. and Emerenciano, M. G. C., 2020. *Tenebrio molitor* meal in diets for Nile tilapia juveniles reared in biofloc system. Aquaculture, 519, 734763.
21. Yan, Y. and Liang, Y., 2004. Energy flow in *Branchiura sowerbyi* (Oligochaeta: tubificidae) in a shallow macrophyte-dominated lake, Biandantang Lake. Chin. J. Oceanol. Limn. 22(4), 403-407.
22. Zaki, M. A., Alabssawy, A. N., Nour, A. E. A. M., El Basuini, M. F., Dawood, M. A., Alkahtani, S. and Abdel-Daim, M. M., 2020. The impact of stocking density and dietary carbon sources on the growth, oxidative status and stress markers of Nile tilapia (*Oreochromis niloticus*) reared under biofloc conditions. Aquacult Rep 16, 100282.
